# Potentials of Natural Preservatives to Enhance Food Safety and Shelf Life: A Review

**DOI:** 10.1155/2022/9901018

**Published:** 2022-09-23

**Authors:** Ejigayehu Teshome, Sirawdink Fikreyesus Forsido, H. P. Vasantha Rupasinghe, Ebisa Olika Keyata

**Affiliations:** ^1^Department of Food Science and Post Harvest Technology, Wachemo University College of Agricultural Sciences, Hosanna, Ethiopia; ^2^Department of Post Harvest Management, Jimma University, Jimma, Ethiopia; ^3^Department of Plant, Food, and Environmental Sciences, Faculty of Agriculture, Dalhousie University, Truro, Nova Scotia, Canada; ^4^Department of Food and Nutritional Science, Wollega University, Faculty of Agriculture, Shambu, Ethiopia

## Abstract

Food-borne illnesses are a significant concern for consumers, the food industry, and food safety authorities. Natural preservatives are very crucial for enhancing food safety and shelf life. Therefore, this review aimed to assess the literature regarding the potential of natural preservatives to enhance food safety and extend the shelf life of food products. The review paper indicated that natural antimicrobial agents that inhibit bacterial and fungal growth for better quality and shelf life have been of considerable interest in recent years. Natural antimicrobials are mainly extracted and isolated as secondary metabolites of plants, animals, and microorganisms. Plants, especially herbs and spices, are given more attention as a source of natural antimicrobials. Microorganisms used in food fermentation also produce different antimicrobial metabolites, including organic acids, hydrogen peroxide, and diacetyl, in addition to bacteriocins. Products of animal origin, such as tissues and milk, contain different antimicrobial agents. Natural antimicrobials are primarily extracted and purified before utilization for food product development. The extraction condition and purification of natural preservatives may change their structure and affect their functionality. Selecting the best extraction method coupled with minimal processing such as direct mechanical extraction seems to preserve active ingredients. The activity of natural antimicrobials could also be influenced by the source, time of harvesting, and stage of development. The effectiveness of natural antimicrobial compounds in food applications is affected by different factors, including food composition, processing method, and storage conditions. Natural antimicrobials are safe because they can limit microbial resistance and meet consumers' demands for healthier foods.

## 1. Introduction

In recent decades, the growing demand for natural food preservatives has prompted research into their use in preserving perishable foods [1]. Because food-borne infections are a significant issue for consumers, the food industry, and food safety authorities. Natural antimicrobial compounds that suppress bacterial and fungal development for better quality and shelf life. Food-borne pathogens such as *Clostridium botulinum*, *Staphylococcus aureus*, *Campylobacter jejuni*, *Bacillus cereus*, *Listeria monocytogenes*, *Cryptosporidium hominis*, *and Escherichia coli* 0157: H7 are the main concerns for food safety [2].

Consumption of fresh foods such as seafood, meat, and horticultural products has increased due to the need of consumers for convenient, ready-to-eat, or ready-to-cook foods, and the desire to lead a healthy lifestyle. Fruits, vegetables, seafood, and meat generally have a concise shelf life, thus requiring appropriate preservation technologies to extend their shelf lives [3, 4]. Food-borne disease outbreaks are on the rise even in developed countries, with a shift from challenges posed by foods of animal origin to fresh foods such as minimally processed fruits and vegetables. New risks are being encountered because of changes to food production practices, the environment, an increase in the global trade of food, and changes to the genetic characteristics of the relevant pathogenic microorganisms [5]. The rise in the fresh-cut convenient salad market has coincided with an increase in food-borne diseases; precutting of the salad leaves releases nutrients that support microbial growth. The modified atmosphere within the package reduces spoilage by aerobes but enhances the virulence of pathogens like *E*.*coli* 0157: H7 [6]. The limited storage life of these fresh items and their link to food-borne disease outbreaks has resulted in continued commercial pressure to utilize synthetic chemicals as preservatives. As a result, there are calls for chemical additives to be used as preservatives to prevent food spoiling caused by microbes and the lipid oxidation process. Consumers are becoming more aware of the flaws in synthetic antibacterial and antioxidant compounds and their link to human health problems. This is owing to increased complications such as carcinogenicity, teratogenicity, liver, renal, heart, respiratory, or neurological difficulties, and other ailments resulting from artificial components. In this crisis, consumers demand partial or complete removal of chemically synthesized preservatives from foodstuffs, which are used to extend food shelf life [7].

Natural food preservatives are very safe for utilization. Natural food preservatives are usually obtained from plant, animal, and microbial origins [8]. The main application of natural preservatives in the food industry is to prevent the growth of undesirable microorganisms. It is possible to add plant antimicrobials into the product formulation, coat them on the surface of foods, or incorporate them into the packaging material [1]. However, its mechanism of action is also different. Essential oils derived from plants, enzymes obtained from animal sources, bacteriocins from microbial sources, organic acids, and naturally occurring polymers (chitosan) have been used in different food industries [9].

It is critical to promote and understand the current situation regarding the potential of natural preservation to improve food safety and shelf life of fresh, minimally processed, and ready-to-eat foods to improve future research study interventions and solve the problem of food and nutrition insecurity [10]. However, there is a paucity of comprehensive reviews on the potential of natural preservation, particularly antimicrobial and antioxidation agents found in plants and animals, application of natural edible coatings, and factors affecting antimicrobial activities of natural products to improve food safety and shelf life [11]. As a result, the goal of this review was to gather the literature on natural food preservatives' potential and function in improving food safety and shelf life.

## 2. Potentials of Natural Preservatives to Boost the Safety and Shelf Life of Foods

Traditional food preservation methods are ineffective in reducing the spread of food-borne viruses in food items. The growing desire for chemical-free food has paved the way for the use of antimicrobials in the food sector [12]. Antimicrobials are a novel technology used by the food industry to extend the shelf life of food and address quality and safety concerns [13].

### 2.1. Natural Antimicrobial Agents

Primary metabolites serve an essential purpose in the organism, whereas secondary metabolites may be waste products or play an essential role in production. Natural antimicrobials are secondary metabolites with antimicrobial action. Plants (fruits, vegetables, seeds, herbs, and spices), animals (eggs, milk, and tissues), and microorganisms (fungi and bacteria) can all be used to extract them [14, 15]. Secondary metabolites have been discovered to be beneficial substances that act as antimicrobials or disease-controlling agents in plants [16]. Due to the antibacterial ability of secondary metabolites against pathogenic and spoilage microorganisms, they are becoming increasingly important for use in food items [17]. They are believed to be a superior option for food preservation to synthetic preservatives because they have antibacterial and antioxidant characteristics at the same time.

#### 2.1.1. Application of Plant-Based Antimicrobials

Natural antimicrobials can be found in raw vegetables, fruits, and herbs/spices. Fruits and vegetables (garlic, pepper, onion, cabbage, and guava), seeds and leaves (olive leaves, parsley, caraway, nutmeg, fennel, and grape seeds), and herbs and spices (marjoram, basil, oregano, rosemary, thyme, sage, clove, and cardamom) all include natural plant-derived chemicals [18]. Essential oils (Eos) and extracts derived from plants have long been used as food additives to improve taste and impart distinct flavors, and extend the shelf life of foods by preventing rancidity and controlling microbiological contamination. Indeed, these chemicals can limit or impede the growth of harmful bacteria because of their high presence of secondary metabolites, primarily phenolic compounds, iso-flavonoids, terpenes, ketones, aliphatic alcohols, acids, and aldehydes [19].

The antimicrobial activity of plant-derived compounds mainly depends on microorganism type, inoculum size, culture medium, extraction method, and method for antimicrobial activity determination [18]. Plant-based compounds, such as polyphenols, terpenes, and alkaloids, are produced from natural sources. Because of their biocidal effects against bacteria or herbivore repellence, plants have a diverse range of secondary metabolites that protect them from predators and microbial pathogens [20]. The phenolic and polyphenolic groups are two of the significant classes found in secondary metabolite compounds. Flavonoids, quinones, coumarins, phenolic acids, tannins, phenols, flavones, and flavonols are some of the subgroup chemicals important for inhibiting microbial activity. Phenol is a hydroxyl (-OH) group-containing chemical. The amount and locations of phenol groups present in the substance correlate with their relative microbial toxicity.

Many Eos have been classified as generally recognized as safe (GRAS) by the US Food and Drug Administration (FDA) and may be used as food preservatives [21]. Eos is the most crucial phytochemical employed in food preservation [22]. Eos are highly volatile, sweet-smelling molecules with an oily consistency, produced by plants. EOs can be extracted from various plant components, including flowers, seeds, and leaves. Essential oils can be extracted from various portions of aromatic plants using a variety of processes, including distillation, supercritical fluid, and many more. The Eos antibacterial properties are oxygenated terpenoids (such as alcohol and phenolic terpenes). Several hydrocarbons, such as aliphatic, monoterpene, and sesquiterpene hydrocarbons, have characteristics that make them microbially active [20].

Herbs and spices are used as antibacterial agents. Eos extracted from plants, spices, and herbs has a high vapor pressure and can reach microbes via liquid and gas phases [5]. Eos' hydrophobic qualities react with lipids in the microbe's cell membrane, causing the cell wall to disintegrate. As a result, the cell's permeability will grow, and the cytoplasmic membrane will be damaged. There will be cell content leakage and cytoplasm coagulation. As a result, the cell's original structure will be disrupted [23].

Bitencourt et al. [21] found that by employing a two-fold dilution of mint essential oil, the inhibition of *Escherichia coli* and *Salmonella* enteritidis could be resolved using the usual broth dilution approach. The use of an edible coating containing mint essential oil prevented the growth of *Escherichia coli* and *Salmonella* enteritidis; the higher the mint essential oil concentration, the lower the microbiological activity. Matan [24] found that using anise oil, which includes multiple active chemicals including trans-anethole, -trans-bergamotene, and limonene, inhibits bacteria such as *Salmonella typhimurium*, *Staphylococcus aureus*, and *Vibrio parahaemolyticus* was adequate. These bacteria are commonly found in seafood. Furthermore, anise oil may prevent spore germination.

Furthermore, according to Matan [24], cinnamon and clove oil include a variety of active chemicals such as cinnamaldehyde, eugenol, and linalool. As a result, both oils reduce the growth of yeasts and molds, and extend the shelf life of dried fish. According to Rehman et al. [25], citrus peel essential oils have various uses in bread. According to the findings, the oils impacted sensory properties and slowed microbial growth. Spraying peel essential oil has the most inhibitory impact against molds and germs. To extend the shelf life of gluten-free sliced bread, researchers used active packaging using cinnamon essential oil and modified atmosphere packaging (MAP). The active packaging outperformed MAP by increasing product shelf life since it reduced microbial development while retaining the gluten-free bread's sensory qualities [26].

Aronian berry, asparagus, bell pepper, beet, blackberry, blueberry, broccoli, carrot, cucumber, cherry, cranberry, garlic, ginger, grape, red onion, red cabbage, rhubarb, raspberry, pomegranate, spinach, strawberry, and green tea have all been studied for their antibacterial effects [27]. According to the authors, all green vegetables have no antibacterial action against *Staphylococcus epidermidis* and *Klebsiella pneumonia*, while all purple and red vegetables and fruit juices have. Pomegranate juice has reduced *E*. *coli* O157: H7 growth [28].

Fruits, vegetables, nuts, seeds, stems, flowers, and leaves contain saponin and flavonoids [29]. Saponins and flavonoids obtained from plants such as Bersama engleriana (Melianthaceae) have been shown to have antibacterial activity when extracted from roots, stems, bark, leaves, and wood [30, 31]. The antimicrobial activity of thiosulfinate has also been demonstrated against Gram-negative bacteria. Glucosinolates are secondary metabolites found in mustards, cabbage, cauliflower, brussels sprouts, broccoli, kohlrabi, kale, horseradish, and radishes, among other plants [32]. Hydrolysis products of glucosinolates have been shown to exhibit substantial antibacterial activity against Gram-positive bacteria, Gram-negative bacteria, and fungus, either alone or in combination with other substances [32].

Pathogenic bacteria and fungi were tested on olive leaves. The aqueous extract of an olive leaf at 0.6 percent (w/v) destroyed practically all bacterium cells in three hours. However, *Dermatophytes* and *Candida albicans* required 1.25 percent and 15 percent (w/v) olive leaf extract, respectively [33]. Olive leaf extract also has potent antibacterial and antifungal properties [34]. Olive leaves' antibacterial and antifungal properties are attributed to phenolic substances such as caffeic acid, verbascoside, oleuropein, luteolin 7-O-glucoside, rutin, apigenin 7-O-glucoside, and luteolin 4-O-glucoside, according to the authors [34]. As a result, the authors indicated that olive leaf extract could be used as a nutraceutical, particularly as a source of phenolic chemicals.  Table 1 shows some of the beneficial effects of plant-derived extracts in food systems.

#### 2.1.2. Application of Animal-Based Antimicrobials

Animals also contain antibacterial compounds that are safe for humans to consume. Antimicrobial compounds produced from animal sources are employed for various applications, as shown in Table 2. The utilization of chitosan, which is commonly used in the food industry, is cited as an example. Chitosan is a polycationic biopolymer typically found in crustacean exoskeletons such as crabs and lobsters. Because of its features, such as its inability to dissolve in neutral conditions and a higher pH value, chitosan's utility in food preservation is limited [49]. Chitosan is now commonly used in edible coatings and films to reduce water vapor content, limit oxygen transmission, and lengthen the shelf life of fruits. As a result, food spoilage will be avoided [50].

Apart from chitosan, lysozyme, which is found in eggs and milk, can also be utilized as an antibacterial of animal origin and is widely accepted as safe. The lysozyme enzyme, found in eggs, is often used as an antibacterial and preservative in chicken, meat, and fruits. Because of its ability to hydrolyze the *β*-1,4 connection between N acetylmuramic acids and N-acetyl glucosamine at the microbial cell wall, lysozyme possesses antibacterial characteristics [49]. Lysozyme is well-known commercially used to prevent *Clostridium tyrobutyricum*-induced late blowing in semihard cheese. Gram-positive bacteria are frequently susceptible to lysozyme but Gram-negative bacteria are not. A lipopolysaccharide layer on the cell membrane's surface causes this effect [50].

Lactoferrin is one of the natural antimicrobial agents found in mammalian secretions such as saliva, milk, and tears, according to Murdock et al. [51], and it is one of the most effective antimicrobial agents in milk. Lactoferrin will help to reduce the amount of iron in the environment. As a result, the bacteria cell's development will be hampered by this circumstance. Lipopolysaccharides will then be released from Gram-negative bacteria's outer membrane, causing the outer membrane to deform. Pores, or “blebs,” will form as a result. Lactoferrin has been shown to suppress the microbiological activity of *Escherichia coli* and *Listeria monocytogenes*.

Antimicrobial peptides are also naturally found in milk. Lactoperoxidase, for example, is a common enzyme found in milk that has been demonstrated to have potent antibacterial properties against bacteria, fungi, and viruses [45]. Cow milk, ewe milk, goat milk, buffalo milk, pig milk, and human milk all include lactoperoxidase [45]. The dairy industry utilizes the lactoperoxidase system to maintain microbiological purity in cow milk. Lactoperoxidase-mediated food preservation affects Gram-negative bacteria more than Gram-positive bacteria.

The antimicrobial activity of animal lipids against various microorganisms has also been reported [52]. Gram-positive and Gram-negative bacteria and fungi may be rendered inactive by milk lipids [53]. Lipids in food may help prevent pathogenic and spoilage microbes from proliferating in food matrixes. Other components found in animals, such as eicosapentaenoic acid and docosahexaenoic acid, have been shown to have antibacterial properties against Gram-positive and Gram-negative bacteria [53].

#### 2.1.3. Application of Microbial-Based Antimicrobials

Microorganisms such as bacteria, fungi, and mold produce various chemicals that are potentially harmful to other microorganisms. Bacteria that fight other bacteria produce a variety of chemicals. Those active bacteria can thwart and prevent the growth of microbes that can cause food deterioration [54].  Table 3 shows some of the microbial-based preservatives used in the food system. Bacteriocin, a protein molecule, is a crucial component that can function as an antibacterial agent against spoilage or microbial pathogens. Gram-positive and Gram-negative bacteria can create bacteriocins [49]. These proteinaceous molecules permeate the cytoplasmic membrane, allowing intracellular metabolites to flow out. As a result, membrane depletion may be taking place. Other active bacteria, such as reuterin and pediocin, can successfully limit the growth of spoilage germs in addition to bacteriocins [19]. Food-borne pathogens such as *Clostridium botulinum*, *Enterococcus faecalis*, and *Listeria monocytogenes* can be inhibited by bacteriocins. Bacteriocins are also safe to employ in bio preservatives because they are protease-degradable [60].

Nisin is a well-known bacteriocin that is often listed in European food additives and by the FDA in the United States. Nisin is commonly used in cheese and sausage preparation [61]. *Lactococcus lactis* produces nisin, made up of amino acids such as lanthionine, dehydroalanine, and aminobutyric acid [60]. Nisin can inhibit a wide range of Gram-positive bacteria. Nisin will be connected to the microbe's cell membrane, and a pore will form due to the ionic contact with the C-terminus. For example, nisin should be used with chelators such as ethylene diamine tetra-acetic (EDTA) [51]. Nisin is used to make cheese because it inhibits the bacteria *Staphylococcus aureus*, which is found in raw milk [23].

Because of its powerful oxidizing effect on the bacterial cell and destruction of basic molecular structures of cell proteins, hydrogen peroxide has significant antibacterial activity [56]. Lactic acid bacteria (LAB) are the main biocontrol agents used to extend the life of perishable products. It acts as an antimicrobial and a probiotic [62]. The antimicrobial activities of lactic acid bacteria are due to their potential to produce various antimicrobials such as hydrogen peroxide, organic acids, carbon dioxide, bacteriocins, reuterin, and ethanol [60]. Lactic acid bacteria (LAB) are thought to produce hydrogen peroxide as their major metabolite [63]. Another antibacterial molecule produced by heterofermentative lactic acid bacteria (LAB) during fermentation is diacetyl (2,3-butanedione). However, due to its buttery aroma and the high concentration required for food preservation, diacetyl's usage as a food preservative has been limited [64]. Acetaldehyde, produced by heterofermentative LAB, has also been demonstrated to have an antibacterial effect against various pathogenic bacteria.

Lactobacillus reuteri produces reuterin and reutericyclin, both of which are active antimicrobials against Gram-positive bacteria. The antimicrobial activity of Reuterin was discovered against Gram-negative bacteria, yeasts, molds, and protozoa. Reuterin (-hydroxy propionaldehyde) is a nonproteinaceous glycerol metabolite that is water-soluble [65]. *L*. *monocytogenes*, *E*. *coli* O157: H7, *S*. *choleraesuis* subsp. Choleraesuis, *Yersinia* enterocolitica, *Aeromonas hydrophila* subsp. Hydrophila, and *Campylobacter jejuni* have all been found to have high antimicrobial activity [66]. Reuter's antibacterial action against Gram-negative bacteria was not boosted when it was tested in combination with nisin [66].

Pediocin, a heat-stable bacteriocin generated by Pediococcus species such as *Pediococcus acidilactici* and *Pediococcus pentosaceus*, is another heat-stable bacteriocin. Most of these peptides are thermostable and active across a broad pH range (pH 2 to 8). Pediocin, unlike nisin, has a relatively narrow range of activity. Overall, pediocins are active against some *Enterococcus*, *Clostridium*, Lactobacillus, Carnobacterium, Pediococcus, and Leuconostoc and *Streptococcus* species as Leuconostoc and *Streptococcus*, yet they have vigorous antimicrobial activity against *L*. *monocytogenes* [67]. Pediocins have been employed as preservatives in various foods, including cheese and meat-based foods. In this regard, Rodriguez et al. [68] found that pediocin preparations from *Lactobacillus lactis* CL1 and L. lactis CL2 *reduced E*. *coli* O157: H7 counts by 0.83 and 1.66 log units, *S*. *aureus* counts by 0.98 and 0.40 log units, and *L*. *monocytogenes* counts by 2.97 and 1.64 log units, respectively, when compared to control cheese at day 30.

### 2.2. Natural Antimicrobial Agents in Edible Coatings

Some researchers have observed the effectiveness of natural antimicrobial agents in reducing microbial contamination when applied directly to food systems, the rapid dispersion of these agents within the bulk of food, and their putative interactions with food components. On the other hand, their antibacterial activity may decrease after storage, limiting their practical utility in the food business. Recently, edible coatings have been examined as a polymeric matrix for trapping natural antibacterial agents as a possible alternative to overcome these restrictions.

Keep active chemical concentrations at a crucial level for long-term microbial growth inhibition by reducing the diffusion of active chemicals onto food surfaces [49].  Table 4 shows some examples of edible coating applications. Furthermore, compared to direct application, this method can produce a highly localized functional effect without compromising the product's organoleptic qualities [70]. Furthermore, edible coatings can serve as a semipermeable barrier, protecting foods from moisture loss, solute migration, gas exchange, respiration, and oxidative processes [75].

Edible coatings are thin layers made from naturally occurring polymers that are applied to food surfaces using various mechanical methods such as spraying, brushing, and dipping [76], or electrostatic deposition [76, 77]. Overall, coating characteristics (composition, chemical structure, viscosity of coating solutions, coating thickness, and degree of cross-linking), coating, processing conditions (temperature, pH, and type of solvent), and type and concentration of additives all influence the functional properties of edible coatings (emulsifiers, plasticizers, or cross-linking agents). The addition of various bioactive compounds, primarily antimicrobial agents, improved the performance of edible coatings by reducing biochemical deteriorations produced by processing, such as textural disintegration, enzymatic browning, and off-flavor development [78]. Edible coatings are utilized as natural antimicrobial delivery systems to improve the shelf life of perishable foods such as fresh and minimally processed fruits, seafood, poultry, and meat products.

Fernandez-Pan et al. [79] found that whey protein isolate-based coatings enriched with oregano EO at a concentration of 20 g/kg could extend the refrigerated shelf life of chicken breast from 6 to 13 days while keeping total mesophilic aerobic, LAB, and *Pseudomonas* spp counts below the critical microbiological limits set for distribution and consumption. According to Bazargani-Gilani et al. [80], CH-based coatings enriched with 2% Zataria multiflora EO effectively controlled microbial growth, delayed chemical changes, improved sensory attributes, and improved sensory attributes, extending the shelf life of chicken breast dipped in pomegranate juice by 15 days during refrigerated storage.

According to Jasour et al. [81], the lactoperoxidase system (LPS) added to CH coatings can extend the shelf life of trout fillets while maintaining excellent sensory acceptability until the 16th day of storage at 4°C. Asik and Candogan [82] observed that CH coatings incorporating garlic oil were efficient in lowering aerobic bacteria counts and prolonging the refrigerated shelf life of shrimp flesh by 2 days.

### 2.3. Application of Antioxidation Agents

Antioxidant compounds are frequently employed to improve the shelf life of food by avoiding oxidative rancidity, degradation, and food color change. Phenolic compounds, vitamin C, and vitamin E are natural antioxidants that serve as free radical scavengers [83]. Unwanted melanosis on the surface of fresh-cut fruits and vegetables is a common problem for merchants [84]. Fruits and vegetables will take on a new appearance due to these changes. This is because the reaction took place on their surface. Two types of enzymes, polyphenol oxidase (PPO) and peroxidase (POD), are used as catalysts in this reaction. The hydroxylation will occur gradually at first, converting monophenol molecules to diphenols. Lipid oxidation is a concern in poultry, meat, and fish, with most lipid oxidation occurring in the muscle due to the oxidation of myoglobin species and hemoglobin [85].

#### 2.3.1. Antioxidation Agents from Plant Origin

Plants are a good source of antioxidants since they contain various active chemicals. Spices, citrus pulp, peel, and oilseeds are high in antioxidant chemicals. For example, black pepper, turmeric, and garlic can inhibit oxidation qualities in many food systems. Because active components such as lignans, flavonoids, polyphenolics, and terpenoids are present in most spices, they have antioxidant properties [85]. Essential oils have been well-known in recent years for their usage as an antioxidant agent for food preservation due to the presence of antioxidant components. By suppressing the oxidation chain reaction, these chemicals can prevent or delay lipid oxidation in chicken, meat, and fish.

Four types of Thai culinary herbs (holy basil, Vietnamese coriander, turmeric, and green peppercorn) were examined for antioxidant capabilities, according to Nugboon and Intarapichet [86]. Compared to turmeric and Vietnamese coriander, holy basil and green peppercorns have a longer shelf life. However, compared to the control meatballs, which lasted less than six days, all of the meatballs tested with Thai herbs had a longer shelf life.

#### 2.3.2. Antioxidation Agents from Animal Origin

Various natural materials have been revealed to be potential sources of natural food preservation chemicals [15]. Free radical scavenging, bleaching inhibition, and reducing power are essential properties of animal-derived antioxidants. Honey has been used to evaluate these capacities. Active substances in honey include phenolic acids, vitamins, and enzymes. Honey has the most potent antioxidant activity due to its flavonoid concentration. They assume that these chemicals, in addition to vitamins, contribute to the antioxidant capacity of honey samples [87].

Aside from honey, chitosan is the best animal-derived antioxidant [88]. Most research on chitosan's oxidative activity reveals that it can slow lipid oxidation and reduce reactive oxygen species in biological systems and foods. Chitosan's antioxidant activity is based on its ability to scavenge free radicals by donating hydrogen or lone pairs of electrons. Chitosan contains essential chemicals for the antioxidant process, such as hydroxyl groups (-OH) and amino groups (-NH). Due to chitosan's semicrystalline structure and strong hydrogen bonds, it is not easy to break. Chitosan inhibits the radical scavenging activity of 1,1-diphenyl-2-picrylhydrazyl (DPPH), superoxide anion radicals, and hydrogen peroxide [89].

## 3. Conclusion

Researchers and food processors are looking for natural food preservatives with a wide range of antibacterial action. Antimicrobial agents, natural antioxidants, and natural edible coating agents are all essential instruments for protecting foods and other related items from the harmful impacts of bacteria and other spoiling processes. Fruits, vegetables, herbs, and spices are the most commonly utilized plants by the food industry as natural antimicrobials to suppress food-borne viruses and improve food shelf life. Peptides are the most common antimicrobial compounds found in mammals (polypeptides). Microorganisms create a variety of chemicals that may be effective against pathogenic and rotting bacteria. Most antibacterial compounds of microbial origin are generated as end-product metabolites during food fermentation. The antibacterial impact of natural products can be influenced by various parameters, including botanical source, harvesting time, development stage, and extraction method. Food components, processing, and other factors in food applications may modify these natural antibacterial compounds. As a result, more significant concentrations may be required for transport and storage than those employed in laboratory media. On the other hand, the sensory properties of food products may be influenced by adding natural antimicrobials. As a result, developing an optimized combination of low doses of antimicrobial agents that can maintain product safety and extend shelf life while minimizing undesirable flavor and sensory changes associated with the addition of high concentrations of natural antimicrobials is a challenge for the practical application of natural antimicrobials. However, natural antimicrobials are only used in many commercial food products.

## 4. Prospect

Even though a considerable number of natural antimicrobials and antioxidants are nowadays known, only a limited number of natural antimicrobial/antioxidant compounds are currently used in commercial applications due to the higher cost associated with them compared to chemical preservatives. Thus, more research needs to be explored utilizing low-cost production methods of natural antimicrobials and antioxidants. Basically, natural antimicrobials and antioxidants are extracted and tested on food products. An extraction method without toxic solvents, such as direct extraction or subcritical water, seems to be a promising method to avoid safety concerns and minimize environmental fingerprints. Natural antimicrobials appear to be the most promising solution for many food safety and quality concerns. Thus, the future will anticipate more investigation of natural antimicrobials in food systems, especially in minimally processed foods. Many types of natural sources are still not being studied to be used as food preservatives. Future research should aim to alleviate this problem and boost the use of natural preservatives in food systems.

## Figures and Tables

**Table 1:** Antimicrobial effect of plant-derived extracts in the food system.

Antimicrobial compound	Target microorganism	Concentration	Antimicrobial effects	Product (food)	Reference
Satureja horvatii EO	*Listeria monocytogenes*	10 and 20 mg/mL	Total inhibition	Pork meat	Bukvicki et al. [35]
Thyme EO	*L*. *monocytogenes*	0.8% and 1.2%	Reduction of viable count below 2 logs (CFU/g) from day six until the end of storage	Minced fish meat	Pellegrino and Tirelli [36]
Oregano and cinnamon cassia Eos	*L*.*monocytogenes*	500 ppm	The growth rate was reduced by 19 and 10 percent with oregano and cinnamon cassia EOs, respectively	Ham	Dussault et al. [37]
Bay leaf EO	Coliforms	0.1 g/100 g	A 2.8 log reduction in total coliforms count on day 12	Fresh Tuscan sausage	da Silveira et al. [38]
Vervain Eos	Monilinialaxa, *M*. *fructigena*	1000 ppm	Reduction of brown rot lesions diameter	Peaches	Elshafie et al. [39]
Thyme Eos Lemon EO	*E*. *coli* O157: H7	500 ppm75 *μ*L/L + thermal treatment at 54°C for 10 min	A 5-log reduction in the initial population	Apple juice	Espina et al. [40]
Lemon EO	*E*. *coli* O157: H7	0.1 mL/100 g	A 1.7 log reduction in *E*. *coli* O157: H7	Chocolate	Kotzekidou et al. [41]
Olive leaves extract	Total viable count	2% (w/v)	A 2-log reduction in the initial population	Raw peeled undeveined shrimp	Ahmad et al. [7]
Chestnut inner shell extract	*Campylobacter jejuni*	2 mg/g	Total inhibition of C. jejuni at an inoculum level of 3 logs (CFU/g)	Chicken meat	[42]

**Table 2:** Antimicrobial substances derived from animal sources and uses.

Antimicrobials	Source	Food bio-preservation	References
Lysozyme	Naturally found as part of a living organism for defence.	Used as antimicrobials in dairy foods and inhibits Gram-positive bacterial species	[43]
Lactoferrin	A type of natural protein seccteted in milk, especially in the whey part.	Antimicrobial activity because of its iron binding property, as well as its antibacterial potency it inhibits *B*. *stearothermophilus*, *L*. *monocytogenes*, *E*. *coli*, and *B*. *subtilis*	[44]
Lactoperoxidase	An antimicrobial system that originated from milk	Effective against gram-negative bacteria	[45]
Ovotransferrin	Produced by hydrolysis of natural proteins.	Inhibits bacterial growth due to iron deprivation	[46]
Protamine	A type of protein found in the sperm of fish (salmon and other species of fish) and birds.	Used as antimicrobial properties inhibits the Gram-positive, as well as Gram-negative bacterias and some species of fungi used as a preservative in a wide variety of foods ranging from confection items to fruits and rice	[19]
Pleurocidin	An antimicrobial peptide secreted in the skin of winter flounder	Inhibits various species of fungus and bacteria including L. monocytogenes, *E*. *coli* O157: H7, V. Parahemolyticus S. *cerevisiae,* and P. expansum	[47]
Chitosan	P produced from chitin for commercial purposes and extracted from exoskeletons of arthropods and crustaceans	Used as antibacterials and antifungals inhibits the growth of *B*. *cereus*, *S*. *typhimurium*, *S*. *aureus*, *L*, *monocytogenes, and Shigella dysenteriae*	[48].

**Table 3:** Antimicrobial substances derived from the bacterial cell.

Antimicrobials	Source	Food bio-preservation	References
Organic acids	Main end products of fermentation.	Decrease the pH of the surrounding environment, creating a selective barrier against nonacidophiles. Lactic acid exerts an antimicrobial effect by disruption of the cytoplasmic membrane and interference with membrane potential.	[55]
Carbon dioxide	Produced by fermentation of sugar by-products using heterofermentative lactic acid bacteria	It creates an anaerobic creation of anaerobic conditions it has antagonistic effects on aerobic bacteria and produces carbonic acid.	[19]
Diacetyl (2,3-butanedione)	A type of low molecular weight compound produced as a metabolic by-product of lactic acid bacteria	Inhibits both Gram-positive and Gram-negative bacterias including *Bacillus cereus*, *Staphylococcus aureus*, *Escherichia coli*, *Salmonella anatum*, *Listeria monocytogenes Yersinia*, and *Aeromonas*.	[19]
Hydrogen peroxide	Produced by LAB in the presence of oxygen and action of flavoprotein oxidases or NADH peroxidase.	The antibacterial effect through oxidative damage of proteins and increase of membrane permeability	[56]
Reuterin	A kind of antimicrobial compound with low molecular weight; it is produced by *Lactobacillus reuteri* by anaerobic metabolism of glycerol.	Effective against *Listeria monocytogenes*	[57]
Reutericyclin	Produced and isolated from *Lactobacillu reuteri*	Antibacterial and it effectively inhibits Gram-positive bacteria including *B*. *cereus*, *B*. *subtilis*, *E*. *faecalis Listeria innocua S*. *aureus*, and *Clostridium difficile*.	[58]; [19]
Nisin	Nisin synthesized by some strains of *Lactococcus lactis* is a heat-stable bacteriocin peptide	Nisin inhibits target cells via specific binding to the cell wall precursor lipid II, followed by the formation of pores in the bacterial cell membrane and subsequent loss of intracellular constituents	[59]

**Table 4:** Application of edible coating to extend the shelf life of foods.

Types of based	Composition	Food additives	Application & results	Reference
Polysaccharides	Chitosan, distilled water, tween 80, and palm stearin	Chitosan (antimicrobial agent)	Chitosan-stearin edible coatings on star fruits (*Averrhoa carambola* L.) can extend the shelf life at low temperatures and maintain their firmness and appearance.	[69]
	Cassava starch, copaiba oil, and distilled water	Copaiba oil (antimicrobial agent)	Coating with cassava starch and copaiba oil on organic strawberries at low temperatures show the lesser counts of mesophilic and psychotropic microorganisms, yeast, and mold.	[70]
	Chitosan, glycerol, tween 80, and distilled water	Chitosan (antimicrobial agent)	The coating based on chitosan-glycerol to delay the “berangan” banana ripening process at ambient air is an effective method	[71]

Polysaccharide & protein	Whey protein, soy protein, alginate, carrageenan, glycerol, and distilled water	Alginate and sunflower oil (antioxidation agent)	Effect of using different edible coatings on fresh-cut apples to extend their shelf life. Soy from plant protein and whey from milk protein are used for coating, and the addition of sunflower oil helps to improve the quality of the fruit.	Ghavidel et al. [72]
Protein	Protein, lauric acid, propylene glycol, and distilled water	—	Using soy protein-based is improving the shelf life and overall quality of minimal processed jujubes.	[73]
	Gum acacia, garlic, and cinnamon	Garlic, cinnamon (antimicrobial agent and anti-oxidation agent	Gum acacia edible coating incorporated with garlic and cinnamon as a natural preservative for meat and fish shows garlic and cinnamon can be used as antimicrobial and antioxidant agents. The shelf life is extended until three weeks and the microbial presence decrease week-wise.	Rakshit and Ramalingam [74]
